# Gadgets Meet Artefacts: Aligning Heyes’s Cultural Evolutionary Account With the Archaeological Record

**DOI:** 10.1177/17456916231187405

**Published:** 2023-08-02

**Authors:** Ross Pain

**Affiliations:** Department of Philosophy, University of Bristol

Heyes (2022) presents a compelling alternative to nativist norm psychology. According to the latter, the psychological processes that explain normative behavior are domain-specific and genetically inherited. In Heyes’s view, those processes are either implicit, domain-general, and genetically inherited, or explicit, domain-specific, and culturally inherited.

Heyes’s focus is on the evidence from human ontogeny; here I want to look at the evidence from human evolution. The ontogenetic claims made by the gadgets account have important phylogenetic implications. Specifically, I want to look at how a cultural evolutionary account of norms aligns with archaeological data; in particular, the stone-tool record. At the outset, there are two important points to be made here.

The first point is an evidential claim: The stone-tool record offers an evidential trajectory, beginning 3.3 million years ago, of the evolution of normative behavior and psychology. On one hand, the production of the earliest stone tools—such as the Oldowan (2.6–1.7 million years ago)—plausibly required no rich normative cognitive processes, whether culturally or genetically inherited (e.g., Snyder et al., 2022). On the other hand, few would deny that later tool types—such as Solutrean points from 22,000 years ago—required explicitly represented norms for teaching and transmission, and hence the cognitive ability to process those norms (see Schmidt, 2015, for an overview of the behavioral and social demands of Solutrean point making). So somewhere between the Oldowan and the Solutrean, the psychological processes driving normative behavior evolved. The stone-tool record is thus evidence of normative psychology because the emergence of more complicated technologies is evidence of our ability to cognitively process normative information. It is no accident that the core features of normative behavior that Heyes singles out—compliance, enforcement, and commentary—likewise describe key aspects of the kind of social learning and teaching processes required for the production and intergenerational transmission of toolmaking abilities.

The second point is a causal claim: Stone tools do not only demonstrate our normative capacities; they plausibly played a causal role in the evolution of those capacities. Birch (2021) offers the most developed account of this kind. In his view, cognitive capacities that had evolved for the guidance of skilled action in toolmaking provided the platform from which our richer normative capacities emerged. The idea is that internally represented norms of correct performance in manual praxis, regulated by cognitive-control models, could be elaborated into the normative cognition displayed by modern-day humans. His account points to a tool-norms coevolutionary hypothesis: The ability to make tools supports the evolution of norms, and the evolution of norms supports the ability to make more sophisticated tools.

With these ideas in mind, we can turn to the question of human cognitive phylogeny and the archaeological record. A key question for Heyes’s framework is the following: When in human evolutionary history did the explicit processes driving commentary behavior emerge? This question is key, because the answer tells us when the norm gadget evolved—and if we know the chronology we can start to pin down other details, such as the hominin species involved, their sociobiology, ecology, and so on.

So when did tools requiring explicit commentary for their production emerge? There will be a range of different views on this question, but I suggest the following answer: Cognitive gadgets emerged at some point during the Acheulean. The Acheulean began 1.7 million years ago and was replaced by the Middle Stone Age around 300,000 years ago. Although the uniformity of the Acheulean is often emphasized, there is notable change in the techniques required to produce tools of the late Acheulean (from 600,000 years ago). In particular, early Acheulean tools were created solely using other stone tools, whereas late Acheulean tools were often shaped and thinned using a tool made of antler, bone, or wood. The upscale in behavioral complexity across the Acheulean has been quantified by Stout (2011). It is plausible that early stages of the industry did not require explicit processes and commentary behavior (though they may have). However, given the behavioral complexity involved, this becomes much less plausible in the late Acheulean (e.g., Shipton, 2019). This suggests that norm gadgets emerged in *Homo heidelbergensis* and perhaps late variants of *Homo erectus*. The brief analysis offered here points to important further questions: For instance, do the explicit processes driving commentary behavior require language, or a protolanguage? If so, what does Heyes’s account of norms imply for her gadget account of the evolution of language (2018, pp. 169–196)?

A final point concerns the following question: Did gadgets evolve suddenly or gradually? In some of her previous writing, Heyes has suggested that gadgets evolved suddenly (2018, pp. 210–213). I have argued this is a mistake and that cultural evolutionary psychologists should be working to develop a gradualist account of the evolution of gadgets. This is for two related reasons. First, gadgets plausibly have a deeper history than Heyes has previously suggested (Pain, 2023). Second, there are concerns with the notion of major transitions, both in the record itself (Clark, 2009; Clark & Riel-Salvatore, 2006; Straus, 2009) and with the cognitive, behavioral, and social transitions that archaeological transitions are supposed to signal (Pain, 2019; Shea, 2011). In short, the archaeological record does not support the view that gadgets emerged suddenly. I believe Birch (2021) offers the gadgets account a promising evolutionary framework. Birch (2021, pp. 10–18) outlines a gradualist model of how the implicit norms of correct performance in manual skill might have been elaborated, via explicit norms of teaching, into richer normative notions, such as fairness and shame. His framework offers a route to understanding how the explicit processes driving commentary behavior might have emerged incrementally from the implicit processes underlying compliance and enforcement. This, I suggest, is the right way of thinking about the evolution of cognitive gadgets.

## Transparency

*Action Editor:* Daniel Kelly

*Editor:* Interim Editorial Panel
